# Incidental Chylous Ascites at the Time of Cesarean Section

**DOI:** 10.1155/2015/530210

**Published:** 2015-06-15

**Authors:** Kida A. Thompson, Antoun Al Khabbaz

**Affiliations:** ^1^Department of Family Medicine, University of Illinois College of Medicine at Rockford, 1200 East State Street, Rockford, IL 61104, USA; ^2^Department of Obstetrics and Gynecology, University of Illinois College of Medicine at Rockford, 1601 Parkview Avenue, Rockford, IL 61107, USA; ^3^Crusader Community Health, 1100 Broadway, Rockford, IL 61104, USA

## Abstract

Chylous ascites has multiple etiologies including malignancies, liver cirrhosis, intraperitoneal infections, and trauma. It is rarely reported in pregnancy. We report a case of chylous ascites noted at the time of cesarean section performed at 35 weeks of gestation on a patient with preeclampsia and suspected placental abruption. The diagnosis and treatment of chylous ascites as well as the pregnancy outcome are presented. A literature review of chylous ascites in pregnancy is discussed as well.

## 1. Introduction

Chylous ascites is the extravasation of lymphatic fluid into the peritoneal cavity. It is usually milky in appearance and high in triglyceride content. It has multiple etiologies including trauma to the abdomen, malignancies, and intraperitoneal infections. It has also been reported as a postoperative complication [1]. Chylous ascites has rarely been associated with pregnancy; not often does an obstetrician encounter chylous ascites. Only six cases of chylous ascites in pregnancy have been published, of which only one had no clear underlying etiology.

## 2. Case

A 29-year-old primigravida patient was admitted to the labor and delivery suite at 34 weeks of gestation with a diagnosis of preeclampsia. Her systolic blood pressures ranged between 140 and 156 mmHg and her diastolic blood pressures ranged between 91 and 108 mmHg. Her workup revealed hyponatremia and hypoalbuminemia with no other abnormalities. She was observed for signs of severe preeclampsia. At 35 weeks of gestation, the patient started having vaginal bleeding and mild abdominal pain. Placental abruption was suspected. The patient was remote from delivery. She was counseled and consented to undergo a primary cesarean section.

**Figure 1:**
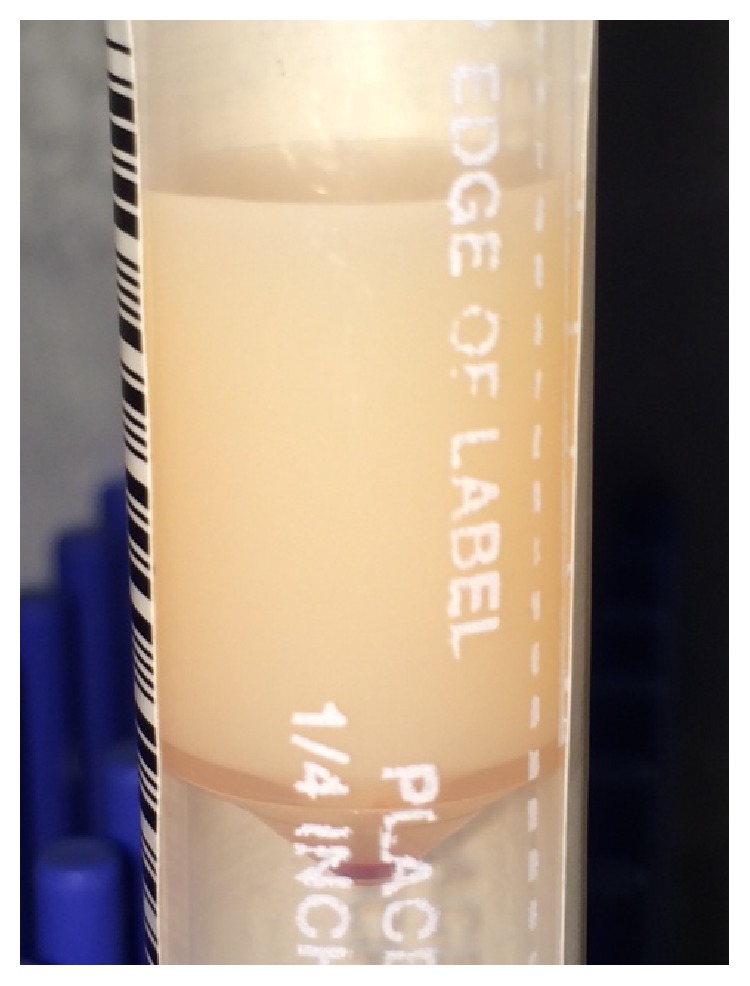
A sample of the chylous ascitic fluid.

At the time of cesarean section and upon entering the peritoneal cavity, a copious amount (around 800cc) of milky peritoneal fluid was evacuated (Figure 1). The exact nature and etiology of the fluid were unknown to us at that time. Surgery was completed. A live male newborn was delivered with a birth weight of 2020 g. Apgar scores were 8 at 1 minute and 9 at 5 minutes. Amniotic fluid was clear and an estimated 20% placental abruption was clinically evident. A general surgeon was called for intraoperative consultation. The abdomen was explored by the general surgeon; no abnormalities were noted in the cecum, small bowels, omentum, uterus, ovaries, or fallopian tubes. A Jackson-Pratt (JP) abdominal drain was placed at the end of the procedure.

The patient was started on intravenous antibiotics for the possibility of intraperitoneal infection. Culture of the peritoneal fluid was negative. Triglyceride level of the peritoneal fluid was 863 mg/dL (diagnostic of chylous ascites). The patient had no clinical evidence of pancreatitis.

The intravenous antibiotics were discontinued after peritoneal culture results were available. The drained peritoneal fluid became progressively serous. The JP drain was removed on the third postoperative day after drainage became clinically insignificant.

The patient received magnesium sulfate therapy for seizure prophylaxis for 24 hours postpartum. She remained hemodynamically stable and regained her bowel function. She was discharged from the hospital on the fourth postoperative day.

A CT scan of the abdomen and pelvis was performed at 6 weeks postpartum and showed no evidence of ascites and no abdominal masses.

## 3. Comment

Ascites is the accumulation of fluid in the peritoneal cavity. There are many different types of ascites including pancreatic, bilious, malignant, chylous, and tuberculous. Chylous ascites is usually due to the obstruction of lymphatic channels and leakage of lymphatic fluid into the peritoneal cavity via a fistula from dilated retroperitoneal lymphatics [1]. Chylous fluid is high in triglyceride content and the diagnosis is usually made when the concentration is greater than 200 mg/dL.

Chylous ascites can be due to a myriad of clinical conditions. Malignant tumors account for the majority of cases of chylous ascites, with lymphoma being the most commonly cited tumor [1, 2]. Mesenteric tumors are also a common etiology of chylous ascites. Liver cirrhosis and abdominal trauma are other reported causes. Infections including disseminated tuberculosis and lymphatic filariasis are common causes in underdeveloped countries [1]. Postoperative development of chylous ascites has also been reported especially following abdominal surgery and pelvic lymph node dissection [1].

In pregnancy, chylous ascites is an extremely rare occurrence. After a review of the literature, we found six reported cases. Habek et al. [3] described a case of chylous ascites in pregnancy. The patient was previously diagnosed with a chylothorax secondary to pulmonary tuberculosis in childhood. Further evaluation revealed congenital lymphangiectasia. Chuang et al. [4] reported a case of an acute abdomen in a pregnant woman who was found to have chylous ascites attributed to severe pancreatitis. Another case of chylous ascites attributed to pancreatitis was reported by Liu et al. [5]. The diagnosis was made by exploratory laparoscopy due to acute epigastric pain. The ascites resolved postoperatively. Sun et al. [6] described a patient with chylous ascites found at the time of cesarean section. The patient was later diagnosed with a mesenteric tumor by a spiral CT of the abdomen and pelvis. She subsequently underwent surgical excision of the mass with a final histologic diagnosis of mesenteric fibromatosis. Kondrat'ev [7] reported a case of chylous ascites related to volvulus of the small intestine. More recently, Babic et al. [8] reported a case of spontaneous chylous ascites in a woman with morbid obesity, in which ascites was discovered at the time of the cesarean section. Complete resolution of the ascites occurred, with no malignancies noted on CT of her abdomen and pelvis postpartum.

After reviewing the above mentioned cases, we noted that none of the patients was reported to have preeclampsia. In our case, preeclampsia might have played a role in the formation of ascites. As a matter of fact, the development of ascites has been linked to preeclampsia in multiple published studies [9, 10]. In the context of preeclampsia, ascites is usually due to hypoproteinemia and a low albumin/globulin gradient resulting in a low intravascular oncotic pressure [11].

In our case, we postulate that the chylous ascites was due to the rupture of pelvic lymphatic vessels after compression by the enlarged gravid uterus. Preeclampsia probably contributed to the volume of the ascites. Pelvic congestion associated with pregnancy could have played a role as well.

The decrease and resolution of the ascitic fluid postpartum paralleled the involution of the uterus in the puerperal period and the improvement of the preeclamptic state.

## 4. Teaching Points/Conclusion

Chylous ascites is a very rare clinical finding in pregnancy. A workup should be conducted to rule out the potential causative conditions. Chylous ascites in pregnancy without an underlying cause seems to resolve spontaneously with the involution of the uterus in the postpartum period. The use of an abdominal JP drain is helpful in the immediate postoperative period. Preeclampsia might be a contributing factor to the volume of ascites present.
